# The Relationship Between Autophagy and Brain Plasticity in Neurological Diseases

**DOI:** 10.3389/fncel.2019.00228

**Published:** 2019-05-24

**Authors:** Man-Man Wang, Ya-Shuo Feng, Si-Dong Yang, Ying Xing, Jing Zhang, Fang Dong, Feng Zhang

**Affiliations:** ^1^Department of Rehabilitation Medicine, The Third Hospital of Hebei Medical University, Shijiazhuang, China; ^2^Department of Spine Surgery, The Third Hospital of Hebei Medical University, Shijiazhuang, China; ^3^Department of Clinical Laboratory Medicine, The Third Hospital of Hebei Medical University, Shijiazhuang, China; ^4^Hebei Provincial Orthopedic Biomechanics Key Laboratory, The Third Hospital of Hebei Medical University, Shijiazhuang, China

**Keywords:** autophagy, brain plasticity, neuroprotective effect, signal pathway, neurological disease

## Abstract

Autophagy, a catabolic degradation system, is utilized for destroying and recycling the damaged or unnecessary cellular components. Brain plasticity refers to the remarkable characteristics of brain neurons that change their structure and function according to previous experience. This review was performed by searching the relevant articles in databases of SCIENCEDIRECT, PUBMED, and Web of Science, from respective inception to January 2019. Here, we review the neuroprotective effect of autophagy in neurological diseases and the mechanism of autophagy in brain plasticity. Moreover, the mechanism of autophagy in the process of brain plasticity can provide the possibility for the development of new treatment methods in the future, thus benefiting patients with neurological diseases. In summary, autophagy and brain plasticity play important roles in neurological diseases.

## Introduction

Autophagy is a lysosome-reliant degradation mechanism that regulate many biological courses, such as neuroprotection and cellular stress reactions (Shen and Ganetzky, 2009). There are different kinds of autophagy in most mammalian cells, and each type of autophagy performs very specific tasks in the course of intracellular degradation (Tasset and Cuervo, 2016). The autophagy-lysosomal pathway is a main proteolytic pathway, which mainly embraces chaperone-mediated autophagy and macroautophagy in mammalian systems (Xilouri and Stefanis, 2010). Macroautophagy, as a lysosomal pathway in charge of the circulation of long-lived proteins and organelles, is mainly considered as the inducible course in neurons, which is activated in conditions of injury and stress (Boland and Nixon, 2006). Coupled with macro-autophagy, chaperone-mediated autophagy (CMA) is crucial for maintaining intracellular survival and homeostasis via selectively reducing oxidized, misfolded, or degraded cytoplasmic proteins (Cai et al., 2015).

**FIGURE 1:**
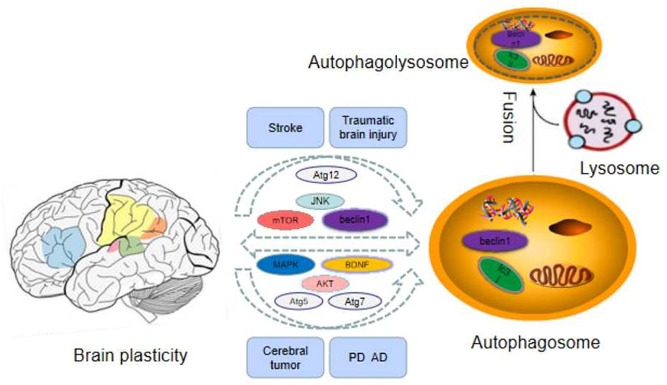
The related important factors of autophagy and brain plasticity.

The plasticity of the central nervous system(CNS) can be regarded as changes of functional interaction between different types of cells, astrocytes, neurons, and oligodendrocytes (Aberg et al., 2006). The mature brain, as a highly dynamic organ, constantly alters its structure via eliminating and forming new connections. In general, these changes are known as brain plasticity and are related to functional changes (Viscomi and D’Amelio, 2012). Brain plasticity can be divided into structure plasticity and function plasticity. The structural plasticity of the brain refers to the fact that the connections between synapses and neurons in the brain can be established due to the influence of learning and experience. It includes the plasticity of synapses and neurons. Synaptic plasticity refers to the changes of pre-existing relationship between two neurons including structure and function alteration (De Pitta et al., 2016). Synaptic plasticity is considered as the representative of cellular mechanisms of memory and learning. Mitochondria are related to the modulation of complicated course of synaptic plasticity (Todorova and Blokland, 2017). For a long period, synaptic plasticity has been considered as a neuronal mechanism under the regulation of neural network action (Ronzano, 2017). Recent data indicate that autophagy is a homeostatic mechanism which is compatible with the microenvironment of the synapse, with the purpose of serving local functions linked with synaptic transmission (Todorova and Blokland, 2017). Neuronal plasticity is maintained by the fine modulation of organelle biogenesis and degradation and protein synthesis and degradation to assure high-efficiency turnover (Viscomi and D’Amelio, 2012). Protein degradation plays an important role in the course of synaptic plasticity, but the involved molecular mechanisms are unclear (Haynes et al., 2015). Therefore, Autophagy is a quality control mechanism of organelles and proteins in neurons, which plays a crucial role in their physiology and pathology (Viscomi and D’Amelio, 2012). In a word, there is a close relationship between autophagy and brain plasticity, and the related mechanisms are summarized in this review paper (as Table 1 and Figure 1 demonstrate).

**Table 1:** The summary for involved signal pathways for the neuroprotective effect via regulating autophagy.

References	Pathway	Neuroprotective effect via activating / inhibiting autophagy	Diseases
Wang et al., 2012	TSC2-mTOR-S6K1	Activating	Cerebral ischemia.
Guo et al., 2014	AMPK/mTOR and JNK pathways	Inhibiting	Ischemia-reperfusion injury
Chen et al., 2018	mTOR/p70S6K	Inhibiting	Ischemia/reperfusion injury
Jiang J. et al., 2018	mTOR/Ulk1	Inhibiting	Ischemic stroke
He et al., 2018	PI3K/AKT	Activating	Traumatic Brain Injury
Feng et al., 2017	PERK and IRE1	Inhibiting	Ischemic stroke
Shen et al., 2017	AMPK	Activating	Stroke
Wang et al., 2014	MiRNA-30a	Activating	Ischemic stroke
Zhou et al., 2011	Gsk-3	Activating	Ischemic brain injury
Zhang Y. et al., 2016	MiR-214-3p	Inhibiting	Sporadic Alzheimer’s disease
Hu et al., 2017	ATG5	Activating	Parkinson’s Disease

## The Neuroprotective Effect of Autophagy in Neurological Diseases

Autophagy is involved in the occurrence and treatment for a series of neurological diseases. However, there are only sporadic reports for the relationship between autophagy and some types of the neurological diseases, which have not been accumulated enough to be reviewed. Therefore, in this review, we summarize the relationship between autophagy and brain plasticity in stroke, traumatic brain injury, cerebral tumor, and neurodegenerative diseases.

### Autophagy and Stroke

Autophagy plays different roles in various conditions, and both autophagy activation and autophagy inhibition could exert neuroprotective effects in the process of stroke.

### The Neuroprotective Effect of Autophagy Activation in Stroke

The co-modulation of autophagy and apoptosis is involved in ischemic stroke (IS)-induced injuries, and apoptosis and mitochondrial autophagy play an important role in this process (Guo Y. et al., 2017). RIPer (Remote ischemic perconditioning) has obvious neuroprotective effect on cerebral ischemia reperfusion injury in rats, and the autophagic lysosomal pathway is activated by RIPer. Autophagy activation promotes the neuroprotective effect of RIPer on focal cerebral ischemia in rats (Su et al., 2014). Liu et al. (2018b) report that activation of autophagy flux in astrocytes might conduce to neural recovery mechanisms and endogenous neuroprotective following stroke. Nampt promotes neuronal survival via inducing autophagy by modulating the TSC2-mTOR-S6K1 signaling pathway in a SIRT1-reliant manner during cerebral ischemia (Wang et al., 2012).

### The Neuroprotective Effect of Autophagy Inhibition in Stroke

There is increasing evidence that autophagy dysfunction leads to the accumulation of damaged organelles and/or abnormal proteins. This accumulation is associated with synaptic functional disorder, neuronal death, and cellular stress (Xilouri and Stefanis, 2010). RIPreC (Remote ischemic preconditioning) + IPOC(ischemic post-conditioning) reduced the plasma HMGB1 level to exert its neuroprotective effect on cerebral ischemia reperfusion injury by suppressing the autophagy process (Wang et al., 2016a). SMXZF, which is a kind of compound extracted from Chinese traditional medicine, plays a neuroprotective part in focal ischemia-reperfusion injury, which might be related to the autophagy inactivation via AMPK/mTOR and JNK pathways (Guo et al., 2014). Guo D. et al. (2017) demonstrate for the first time that suppression of MALAT1 reduces beclin1-reliant autophagy via regulating the expression of mir-30a in cerebral IS, thereby reducing neuron cell death. Moreover, autophagy is regulated by mammalian target proteins in the PI3K/AKT/mTOR/p70S6K signaling pathway (Fan et al., 2015). RIPostC could suppress autophagy via activating the mTOR/p70S6K signaling pathway, thus reducing the brain I/R damage (Chen et al., 2018). Vitexin regulated autophagy dysfunction to alleviate MCAO-induced cerebral IS through mTOR/Ulk1 pathway (Jiang J. et al., 2018). The increasing evidence indicates that the AMPK-mTOR signaling pathway mediates the autophagy activity by the coordinated phosphorylation of ULK1 (Wang et al., 2018). Zheng et al. (2012) have demonstrated that NAD(+) administration reduced ischemic brain injury at least partly via inhibiting autophagy. LncRNA H19 inhibits autophagy through dusp5-erk1/2 axis. Blood samples from patients with IS showed that H19 gene mutation increased the risk of IS. LncRNA H19 could be a novel therapeutic target for IS (Wang et al., 2017a).

In summary, different studies obtained various results for the role of autophagy in the process of stroke, and further studies are required to explore the relationship between autophagy and stroke.

### Autophagy and Traumatic Brain Injury

Similar to the relationship between autophagy and stroke, the relationship between autophagy and traumatic brain injury is also not simple. Both inhibition and activation of autophagy could exert neuroprotective effects following the occurrence of traumatic brain injury.

#### The Neuroprotective Effect of Autophagy Activation in Traumatic Brain Injury (TBI)

The promotion of autophagy and neuronal apoptosis are related to the secondary neural injury after Traumatic Brain Injury (TBI). Sevoflurane post-conditioning regulates autophagy through PI3K/AKT signaling, which alleviates the TBI-triggered neuronal apoptosis (He et al., 2018). Zhang L. et al. (2016) for the first time reveal that FTY720 plays a neuroprotective role following TBI, at least partially through the activation of the PI3K/AKT pathway and autophagy. In addition, Melatonin promotes autophagy, and suppresses mitochondrial apoptosis pathway, thus alleviating secondary brain injury of mice following traumatic brain injury (Ding et al., 2015). RIPoC alleviates brain IR injury via activating AMPK-reliant autophagy (Guo et al., 2018). Calcitriol treatment promotes the expression of VDR protein and alleviated the neural defect in the TBI model of rats. Its protective effect might be related to the decrease of apoptosis and the recovery of autophagy flux in the cortex area of rat brain (Cui et al., 2017). HS (heat stroke) can lead to brain injury via impaired autophagy flux and lysosomal dysfunction, and HA (heat acclimation) has a protective exerts neuroprotection on HS-induced brain injury through the mechanism of autophagy - lysosomal pathway (Yi et al., 2017).

#### The Neuroprotective Effect of Autophagy Inhibition in Traumatic Brain Injury

The autophagy pathway is associated with the pathophysiological reactions following TBI, and suppression of this pathway might contribute to the alleviation of traumatic injury and functional outcome defects (Luo et al., 2011). Melatonin administration before ischemia could notably alleviate brain IR damage by suppressing ER stress-reliant autophagy (Feng et al., 2017). The over-expression of mir-27a may mitigate brain injury by inhibiting Foxo3a-regulated neuronal autophagy after TBI (Sun et al., 2017). Jiang H. et al. (2018) report that the down-regulation of TLR4 improves the neuroinflammatory response and brain injury following TBI by inhibiting astrocyte activation and autophagy induction. Therefore, the effect of activation and inhibition of autophagy on traumatic brain injury should be further clarified before future clinical applications.

### Autophagy and Neurodegenerative Disease

Autophagy is the core regulator of central nervous system senescence and neurodegeneration. The delivery of organelles and toxic molecules to lysosomes by autophagy is critical for the health and survival of neurons (Plaza-Zabala et al., 2017). Most of the neurodegenerative diseases that perplex humans are related to the intracytoplasmic deposition of proteins that tend to accumulate in neurons, and autophagy is a powerful process for removing these proteins (Frake et al., 2015). Autophagy up-regulation is a promising treatment due to its potential to protect cells from the toxicity of accumulated proteins in neurodegenerative diseases (Karabiyik et al., 2017). Autophagy-regulated degradation of synaptic elements sustains synaptic homeostasis but also involves in a mechanism of neurodegeneration (Luningschror et al., 2017). Autophagy is vital for neuronal integrity, and the reduction of important autophagic components results in the structural defects and progressive neurodegenerative changes in pre- and post-synaptic morphologies (Nikoletopoulou et al., 2017). It is demonstrated that autophagy defects arise in the early stage of Alzheimer’s disease (AD) (Li et al., 2017). A considerable amount of evidence indicates that the p38-mitogen-activated protein kinase (MAPK) signaling pathway plays an important part in neurodegenerative diseases and synaptic plasticity (Correa and Eales, 2012). MiR-181a regulates apoptosis and autophagy in PD(Parkinson’s Disease) by inhibiting the p38 MAPK/JNK pathway (Liu Y. et al., 2017). In addition, RhEPO may alleviate hippocampal injury in epileptic seizure rats via regulating autophagy in a time-reliant manner through the S6 protein (Li et al., 2018).

In summary, autophagy has a close relationship with neurodegenerative disease, and activation of autophagy could improve the neurodegenerative changes, which might be a novel target in clinical treatment for such diseases.

### Autophagy and Cerebral Tumor

The role of autophagy in tumor cell survival and death has attracted much attention in recent years (Noonan et al., 2016). As for glioblastoma (GBM), the most lethal tumor of the CNS, there is increasing evidence that the autophagy process is closely related to the tumorigenesis of GBM (Jawhari et al., 2017). Glioblastoma multiform is the most common and invasive primary brain tumor. Due to its adaptive ability of autophagy, it is highly resistant to various treatments (Jawhari et al., 2017). (Gammoh et al. (2016) elucidate that autophagy is the key to the occurrence and growth of GBM, which is an important therapeutic target for the treatment of GBM. The inhibition of autophagy is a promising strategy against GBM, and ATG9 is identified as a new target for hypoxic-induced autophagy (Abdul Rahim et al., 2017). Autophagy played a critical role in the formation of vasculogenic mimicry(VM) via Glioma stem cells (GSCs), which could be used as a therapeutic target for drug-resistant gliomas (Wu et al., 2017). The inhibition of autophagy promotes the anti-tumor activity of ibrutinib in GBM. Wang et al. (2017b) provides important insights into the role of anticancer drugs combined with autophagy inhibitors in the treatment of GBM.

In summary, autophagy provides new therapeutic expectations for cerebral tumor which is a big challenging for human. Therefore, the application of novel therapy for cerebral tumor on the basis of autophagy mechanisms elucidation should be explored in depth.

## The Mechanism of Autophagy in Brain Plasticity

### The Role of Mammalian Target of Rapamycin (mTOR) in the Relationship Between Autophagy and Brain Plasticity

#### Brain Plasticity and mTOR

The mTOR-controlled signaling pathways regulate many integrated physiological functions of the nervous system including neuronal development, synaptic plasticity, memory storage, and cognition (Bockaert and Marin, 2015). Mammalian target of rapamycin, as a protein kinase, is implicated in long-lasting synaptic plasticity and translation control of synapse (Hoeffer and Klann, 2010). It is reported that mTOR modulates many functions in the process of brain development, including proliferation, differentiation, migration, and dendrite formation. Moreover, mTOR plays an important role in the formation and plasticity of synapses (LiCausi and Hartman, 2018). Moreover, the mammalian target of rapamycin complex 1 (mTORC1) is a key modulator for cap-dependent protein synthesis, which is necessary for many forms of long-lasting memory and long-term synaptic plasticity (Santini et al., 2014). Therefore, it is demonstrated that mTOR plays a key role in the process of brain plasticity.

#### Autophagy and mTOR

As a key regulator of autophagy, the mTOR plays an important role in autophagy, translation, cell growth and survival (Hwang et al., 2017). Mammalian target of rapamycin and autophagy are tightly bound within cells, and defects of mTOR and autophagy process might lead to a variety of human diseases (Hoeffer and Klann, 2010). Studies have shown that mTOR is widely involved in autophagy activation and synaptic plasticity (Ryskalin et al., 2018). The mTOR modulates long-lasting synaptic plasticity, memory and learning via regulating the synthesis of dendritic proteins (Liu et al., 2018a). Macroautophagy can degrade organelles and long-lived proteins in case of mTOR inactivation. Synaptic plasticity is further modulated by mTOR and neurodegeneration occurs when macroautophagy is absent (Hernandez et al., 2012). Therefore, macroautophagy following mTOR inactivation at the presynaptic terminal rapidly changes the neural transmission and presynaptic structure (Hernandez et al., 2012). The mechanisms for the target of rapamycin have been involved in modulating neurodegeneration and synaptic plasticity, but the role of mTOR in regulating presynaptic function via autophagy has not been clarified clearly (Torres and Sulzer, 2012).

In summary, there is a close relationship among mTOR, brain plasticity and autophagy. The mTOR related pathways play important role in regulating the process of autophagy and brain plasticity.

### The Related Signaling Pathways of Autophagy and Brain Plasticity

#### The PI3K/Akt Pathway

Autophagy acts as a central mediator of cellular disease and health, and this self-balancing process seems to affect synaptic growth and plasticity in the CNS (Alirezaei et al., 2011). The inhibition of autophagy was necessary for memory improvement and for brain-derived neurotrophic factor (BDNF)-caused synaptic plasticity under the circumstances of nutritional stress, indicating that autophagy was a key component of BDNF signaling pathway, which was critical to BDNF-induced synaptic plasticity (Nikoletopoulou et al., 2017). The BDNF-activated ILK-Akt and PI3K-Akt signaling pathway play an important role in structural synaptic plasticity (Li et al., 2012). The activation of PI3K/Akt pathway might conduce to the memory consolidation and mechanisms of synaptic plasticity via increasing protein synthesis via mTOR pathway and promoting cell survival through FKHR pathway (Horwood et al., 2006). (Xu et al. (2017) reported that the neuroprotection of L-3-n-Butylphthalide (L-NBP) in attenuating learning and memory deficits in mice after repeated cerebral ischemia-reperfusion (RCIR) might be associated with the modulation of the expressions of proteins involved in apoptosis and autophagy and the promotion of Akt/mTOR signaling pathway. Moreover, the activation of PI3 kinase-Akt signaling pathway played an important role in promoting the survival of newly generated granule cells originated during exercise and the related increase of synaptic plasticity of dentate gyrus through an anti-apoptosis function (Bruel-Jungerman et al., 2009). Tetrahydrocurcumin alleviated the damage on neurons against TBI-induced apoptotic neuronal death, possibly through regulating autophagy and the PI3K/AKT pathway (Gao et al., 2016).

Moreover, neuronal stimulation led to NMDA receptor (NMDAR)-dependent autophagy via PI3K-Akt-mTOR pathway suppression, which might work in AMPA receptor (AMPAR) degradation, thus showing autophagy as a promoting factor to brain functions and NMDAR-reliant synaptic plasticity (Shehata et al., 2012). Curcumin exerts neuroprotective role through regulating the PI3K/Akt/mTOR pathway and down-regulating the autophagy activities (Huang et al., 2018). However, the interaction between the PI3K/AKT/mTOR pathway and the autophagy process is complicated, more detailed studies on the mechanism of disease as well as animal and cell models is needed.

#### The MAPK/Erk Pathway

There is increasing evidence that MAPKs can regulate autophagy/macroautophagy (Wang et al., 2016b). MAPK/ErK and p38 play key roles in the strict control of the autophagy process during maturation (Corcelle et al., 2007). In addition, some studies have shown that mitophagy requires MAPKs MAPK1/ERK2 and MAPK14/p38 (Hirota et al., 2015). As a protein kinase, MAPK/Erk is mobilized by neurotrophic factors involved in synaptic plasticity and formation, acting at both the cytoplasmic and nuclear levels (Giachello et al., 2010). The activity-reliant short-term plasticity and formation of these synapses are relied on the MAPK/Erk pathway (Giachello et al., 2010). Recently, there is increasing evidence that Ras and MAPK signaling plays a key role in neuronal function related to synaptic plasticity (Mazzucchelli and Brambilla, 2000).

In summary, the related signaling pathways of autophagy and brain plasticity has not be well clarified, which is worthy of further exploration.

## The Role of Autophagy Mechanism in the Treatment of Nervous System Diseases

The dysfunction of autophagy pathway is related to a variety of neuropathologic conditions, and numerous studies have demonstrated that autophagy is a potential target for pharmacological regulation of neuroprotection (Russo et al., 2015). Autophagy and AMPK play a key role in CSD-induced ischemic tolerance. AMPK-regulated autophagy might be a novel target for stroke (Shen et al., 2017). Wang et al. (2014) report that the suppression of mirna-30a ameliorates ischemic injury by enhancing beclin1-regulated autophagy, which represents a possible therapeutic target for IS. Gsk-3 depressor hinders neuroinflammation following ischemic brain injury via activating autophagy, thereby providing a novel target for the prevention of ischemic brain damage (Zhou et al., 2011). (Peng et al. (2018) definitely indicates that mitofusin 2 mitigates I/R injury primarily by promoting autophagy, providing a potential new strategy for the neuroprotection of brain I/R injury. In addition, miR-214-3p inhibits autophagy and reduces the apoptosis of hippocampal neurons, suggesting that miR-214-3p is a new potential target for sporadic Alzheimer’s disease (SAD) (Zhang Y. et al., 2016). The effect of autophagy related gene 5 (ATG5) in protecting dopaminergic neurons in zebrafish PD model is induced by 1-methyl-4-phenyl-1,2,3, 6-tetrahydropyridine (MPTP) (Hu et al., 2017). Autophagy is closely related to the occurrence and development of various neurological diseases in humans. Therefore, the study of autophagy mechanism has important clinical significance for the diagnosis of diseases and the search for new drug targets.

## Conclusion

Brain plasticity is one of the most fundamental mechanism for neural function recovery following neurological diseases. Autophagy is a crucial lysosome-reliant degradation process that controls various physiological and pathological courses in the brain. The summary for the interaction of autophagy and brain plasticity might provide novel therapy targets for neurological diseases, thus benefiting the patients in clinic.

## Author Contributions

M-MW and FZ conceived the main ideas and wrote the manuscript. Y-SF, JZ, FD, S-DY, and YX searched the references and designed the framework.

## Conflict of Interest Statement

The authors declare that the research was conducted in the absence of any commercial or financial relationships that could be construed as a potential conflict of interest.
